# How decision-styles and cultural orientation influence entrepreneurial and social entrepreneurial intentions: A cross-cultural comparison

**DOI:** 10.3389/fpsyg.2022.988815

**Published:** 2023-01-05

**Authors:** Brandon William Soltwisch, Daniela Dimitrov, Jana Hojnik

**Affiliations:** ^1^Monfort College of Business, University of Northern Colorado, Greeley, CO, United States; ^2^Department of Management, University of Primorska, Koper, Slovenia

**Keywords:** entrepreneurial intentions, cultural orientation, maximizing, satisficing, decision styles, social entrepreneurial intention

## Abstract

This paper investigates how maximizing or satisficing decision styles and cultural orientation influence individuals’ entrepreneurial intentions. With a growing interest in social entrepreneurship, it also measures if these factors encourage individuals to start ventures with a social mission. Two studies are conducted to compare students’ entrepreneurial intentions in the U.S. and in Slovenia. By identifying that maximizing decision styles are associated with an individualistic cultural orientation in both the U.S. and Slovenia, the current study indicates that the maximizing – individualism connection spans national and cultural boundaries. In the U.S. sample, individualism mediated the relationship between decision styles and entrepreneurial intentions, suggesting that in individualistic cultures, such as the U.S., those who maximize their decision efforts and apply a more individualistic cultural perspective are especially inclined to pursue entrepreneurial opportunities. Similarly, individualism mediated the relationship between maximizing and social entrepreneurial intentions in the U.S. sample; suggesting that maximizers who are less individualistic may be more likely to start social enterprises over traditional ventures. Among the Slovenian sample, there was a marginally significant relationship between maximizing and entrepreneurial intentions and no relationship with social entrepreneurial intentions. These cross-cultural differences are discussed in relation to the economic and social conditions in each country.

## Introduction

It is widely accepted that entrepreneurs contribute to economic growth through innovation, new job creation, and competitiveness (Carree and Thurik, 2005; Acs et al., 2012; Stoica et al., 2020), leading to a wealth of research attempting to understand what drives people to pursue new business opportunities. Among a growing list of entrepreneurial trait variables in this research stream, it appears that the way people approach decisions may contribute to one’s desire to start new business ventures. A recent study in the U.S. found that those who seek out additional information and options to find the best alternatives by applying a maximizing decision-making style had greater entrepreneurial intentions than those who settle for good enough choices, known as satisficers (Soltwisch et al., 2022). Behind these increased intentions, the maximizing trait is associated with greater innovativeness and an entrepreneurial orientation that allowed the potential entrepreneurs to identify more potential business opportunities in their environment. The decision strategy has even shown promise for new venture performance as entrepreneurs who maximize appear to build more entrepreneurial and market-oriented business that are more successful (Soltwisch, 2021).

Although this new trait appears promising as a method to study characteristics of entrepreneurs, it remains unknown whether maximizing may relate to entrepreneurial intentions across national contexts, where cultural factors and the availability of entrepreneurial opportunities may change the way people view entrepreneurial decisions. If maximizers are more inclined to identify and pursue new business opportunities in economies that do not foster and support entrepreneurial activity to the same extent as in the U.S., perhaps the search strategy could be a useful tool to help identify potential entrepreneurs and build entrepreneurial skillsets among those considering starting new ventures in countries desiring to create economic vitality through entrepreneurial means. By comparing students in the U.S. and Slovenia, the current study attempts to enhance generalizability of previous findings while understanding how cultural and economic factors may shape a person’s decision to start new business ventures.

As social entrepreneurship becomes an increasingly popular means for governments to fill gaps in the social service sector, the current study attempts to understand how a person’s decision-making style of maximizing or satisficing may relate to their intentions to start social enterprises. Because maximizers seek out the best for themselves and others, it remains unclear whether this tendency to seek out the best may apply to businesses aimed at helping others, such as in the non-profit sector or organizations with a social mission. At the individual level, a person’s cultural perspective toward individualism or collectivism may affect the type of ventures people decide to start (Rantanen and Toikko, 2017); thus, the current study explores how a person’s cultural orientation may affect the type of new businesses they start, whether they choose for profit businesses over social enterprises. By understanding the link between decision-styles, cultural orientation, and entrepreneurial intentions, the current study forms and tests a model of factors that may promote new business ventures across cultural contexts, with the goal of being able to better identify and train those who may be inclined to improve societal outcomes through entrepreneurial means.

1. How does maximizing or satisficing relate to entrepreneurial intentions and social entrepreneurial intentions?
2. How does a person’s decision style relate to their cultural perspective, and does their cultural perspective affect their intentions to start for-profit or social enterprises?
3. Do these relationships hold across national contexts where the nature of entrepreneurial opportunities and cultural environment differ?

The next sections review work in maximizing, satisficing, cultural orientation, and entrepreneurial intentions as the theoretical basis for the research model. The model is tested with a sample of 188 students in the U.S. using multiple regression and mediation analysis. It is predicted that maximizers will display a more individualistic cultural orientation, and that those who apply an individualistic perspective will be more likely to start traditional entrepreneurial enterprises rather than ventures with a social mission. A second study with Slovenian students is conducted to compare how the relationships operate in a more collective culture where opportunities may be more limited. After describing the results, the discussion continues by linking the study’s findings back to specific theoretical and practical applications, providing specific advice for managers and academics on how the model can be used to identify potential entrepreneurs in both collective and individualistic cultures.

## Literature review

### Maximizing and satisficing

Based on Simon’s theory of bounded rationality, satisficing has been reconceptualized by Barry Schwartz et al. (2002) as a measurable trait in which individuals systematically differ in their tendencies to satisfice or maximize, with implications for a variety of personal and work-related behaviors and outcomes (see Schwartz et al., 2002; Schwartz, 2016 for review). The fundamental differences between maximizers and satisficers lies with their decision goals. Satisficers are content with good enough options while maximizers desire to find the best (Schwartz et al., 2002). To find better options, maximizers will continue to search for additional alternatives and compare options even after their criteria have been met. For example, when it comes to purchasing a new phone, maximizers will continue to look at different models to see if a better one may be available even after finding a phone that meets their requirements, say a large screen and good battery life. Alternatively, satisficers may end their search efforts after finding a phone that is “good enough” by obtaining one that meets their minimum criteria. Like personalities, these individual traits have been associated with a variety of personal and work outcomes (see Schwartz et al., 2002; Highhouse et al., 2008; Lai, 2010; Misuraca et al., 2015; Schwartz, 2016 and Misuraca and Fasolo, 2018 for review).

In general, maximizers consider more options, engage in greater comparisons of alternatives, and will spend additional time and effort to find the best (Schwartz et al., 2002; Schwartz, 2016). Due to the contemplative nature of the decision strategy, maximizing has been associated with ruminating over decisions (Schwartz et al., 2002; Paivandy et al., 2008) and counterfactual thinking associated with evaluating more positive and negative outcomes of various alternatives (Polman, 2010; Leach and Patall, 2013). This tireless process sometimes pays off when they find better outcomes. For example, maximizers who search for more job opportunities after graduation land positions with 20% higher starting salaries than those who satisfice (Iyengar et al., 2006). The authors attribute this to their greater efforts to seek out additional opportunities, even one’s that were outside their major area of study. As a strategy, maximizing is more achievement oriented, aimed at finding the best possible outcomes both now and in the future (Bubić and Erceg, 2018). For example, to meet future goals, maximizers have greater savings intentions and will allocate more money toward savings (Zhu et al., 2017).

In the area of work, managers who maximize are more effective in leading their work teams by applying an internal locus of control (Soltwisch and Krahnke, 2017). The combination of searching more extensively together with their feelings of personal responsibility for decision outcomes allows those who maximize to lead their work teams more effectively. The rationale is that those who look for better outcomes may do so because they feel personally accountable for what happens and will put forth greater effort as a result. This aligns with their greater self-efficacy and higher perceived workload (Lai, 2010). Maximizers prefer to control the decision-making process (Sparks et al., 2012), and therefore may prefer entrepreneurship to traditional career alternatives. There is some evidence that they may be more effective entrepreneurs. For example, among entrepreneurs, maximizing decision styles have been associated with building more successful ventures. Interestingly, their maximizing efforts carried over to other aspects of the organization, allowing their new ventures to become more entrepreneurial and market oriented to better serve customers’ changing needs (Soltwisch, 2021). However, after all the hard work to find the best, those who do better often feel worse about their decisions. Psychologically, maximizers exhibit more post-decision regret (Schwartz et al., 2002; Parker et al., 2007; Roets et al., 2012; Bruine de Bruin et al., 2016) and future oriented fear of missing out, or FOMO for short (Müller et al., 2020), as well as general unhappiness, perfectionism, depression and an overall lack of satisfaction and wellbeing in their lives (Schwartz et al., 2002).

This is especially true in cultures that emphasize personal choice as a means for happiness. In one study comparing adults from China, Western Europe, and the U.S., it was found that national culture may attenuate some of the negative psychological outcomes of the maximizing trait. Specifically, they found that maximizers who live in China did not see the same declines in their wellbeing as those living in Western Europe and in the U.S. (Roets et al., 2012). This was attributed to experiencing fewer instances of regret based on the cultural context of living in societies that do not emphasize access to options and personal choice as the primary means of attaining happiness.

This relationship did not pan out in the collective nation of Japan, however, with Japanese maximizers displaying even greater amounts of depression, unhappiness, and lack of satisfaction than those in the U.S. Because the macro-economic conditions in Japan may more closely resemble those in the U.S, the authors attributed these differences to cultural norms around opportunity, noting that “In American cultural contexts, having high standards typically means that a person expects to meet the high standards that the individual sets for him/herself.” (Oishi et al., 2014 p. 19). Alternatively, in Japan, people’s set high standards for themselves but fear that they may not be able to reach their ambitions (Heine et al., 1999). These somewhat contradictory results between China and Japan suggest that perhaps there are personal differences that may affect the way people’s decision styles interact with their social-cultural context. Thus, there is a need to measure the relationship between maximizing or satisficing decision styles and cultural orientation at the individual level to see if the way individuals think about choice relates to how they interact and connect with others. Therefore, the current study aims to answer the call for a greater understanding of the cultural influences on maximizing or satisficing tendencies (Henrich et al., 2010; Oishi et al., 2014).

### Cultural orientation

Based on Hofstede’s cultural dimensions of individualism and collectivism (Hofstede, 1980, 2011) used to describe differences at the national level, individualism and collectivism represent differences in the way people view close knit bounds with others, whether they identify as part of a larger group, how they prioritize group goals over their own, and their desires for personal achievement (Parkes, 2000). Recognizing that individuals who live in national contexts often vary in their cultural perspectives, Triandis and Singelis (1998) developed a measure aimed to inform scholars and professionals on how to recognize differences in personal cultural orientations toward individualism or collectivism (Triandis and Singelis, 1998). If compared to zoology, individualism (I) and collectivism (C) represent the broadest division with a myriad “species” of each, described by culture-specific attributes (Singelis et al., 1995). A more sophisticated method of tempering cultural knowledge with demographic and life experience information is needed to differentiate people within one cultural background from each other. Thus, the attributes that matter to the individual representative of a national sample, are measured by the subjective instrument SINDCOL, and they can be best understood as fluctuating tendencies that might, or might not, be manifested in a particular individual.

Such differences proved significant in predicting specific behaviors and work-related outcomes. For example, individualism is associated with workplace traits of independent decision-making and performance; whereas collectivism is linked to interdependence, comfort, and harmony with others in the workplace (Singelis et al., 1995). For these reasons, individualism has predicted organizational citizenship behavior aimed at increasing status, while collectivism tends to encourage prosocial behavior that benefits others and the organization itself (Lee et al., 2022). Similarly, individualism has been associated with the desire for powerful positions within a company, such as in leadership, while those who take a more collective perspective do not view prestige as important to their career (Parkes, 2000). In addition, collectivistic social behaviors are best predicted by norms, obligations, and duties; whereas, individualists are linked with competition, higher levels of self-reliance (Triandis, 1995), higher level of risk-taking (Chanda and Unel, 2021), and lower level of uncertainty avoidance (UAI; Hofstede, 2011). Individualistic work-related outcomes on an individual level are also innovation, proactive initiatives, resourcefulness, achievement and goal orientation (McClelland et al., 1953; Dimitrov, 2005). Further SINDCOL research (Gürhan-Canli and Maheswaran, 2000) offered value to advertising and consumer behaviors. For example, collectivistic representatives value the superiority of the in-group product.

Among U.S. populations, it was found that individualism was associated with people who were younger; had grandparents from western cultures; have traveled overseas alone or have lived abroad for more than 6 months; have a job that allows one to work without collaboration or in company of others; as well as value their own reasoning, own decision-making, and personal privacy (Triandis and Singelis, 1998). The authors of this personalized cultural measure recognized that the differences could be used to educate individuals on how their own cultural perspectives and the perspectives of people they work with can affect various workplace interactions and behaviors, noting that “training an individual to recognize such variations, within culture, will be of great value” (Triandis and Singelis, 1998, p. 37). The benefits of such knowledge lie in being able to understand the motivation of one’s colleagues in a global business environment. As a result, diversity training in the US was suggested as a direct application of the instrument.

### Cultural orientation and maximizing or satisficing decision-making styles

One of the more profound discoveries related to the way we approach decisions is in how these styles may affect people differently based on cultural contexts. Despite sometimes doing better due to their extensive search strategy, maximizers often feel worse about their decisions, reporting greater instances of regret and depression, as well as being less happy, optimistic, and satisfied in their lives (Schwartz et al., 2002; Iyengar et al., 2006; Parker et al., 2007; Chang et al., 2011; Purvis et al., 2011). Interestingly, the very process that encourages maximizers to explore additional options to find the best becomes a source of unhappiness as they ruminate over past decisions by thinking about what they could have done better and consider their outcomes in relative terms to others, especially with those who are doing better than them (Schwartz et al., 2002; Chan, 2021).

Thus, their notion of what is considered as “the best” is tied to social comparisons. For example, through extensive search, maximizers have been reported to land better jobs after graduation than those who satisfice, yet they feel worse about those better positions because their extensive search process allowed them to see all the opportunities they missed along the way. Additionally, they viewed their results comparatively to others who had been more successful in their job search (Iyengar et al., 2006). This paradoxical finding, that maximizers feel worse despite doing better, appears to depend on cultural factors, as cross-cultural comparisons have found that maximizing has a more negative impact on well-being in societies where choice is abundant, highly valued, and viewed as the primary means for achieving success and happiness, such as in the U.S. (Roets et al., 2012). Alternatively, In China, where choice is more limited and less valued as a means for obtaining happiness through personal achievements, those who maximize did not report the same decreases in well-being associated with ruminating over decisions as those who live in the U.S. or Western Europe (Roets et al., 2012).

Although national culture appears to attenuate some of the negative psychological effects of maximizing, there are heterogeneous cultural orientations that exists within any predominant culture, and many different cultural perspectives within national boundaries; therefore, there has been a call for measuring cultural perspectives at the individual level (Triandis and Gelfand, 1998; Yoo et al., 2011; Kurtiş and Adams, 2013). Support for heterogeneity among cultural values has been found in various studies (Dockens, 2009; Hatt, 2009; Yolles and Fink, 2009; Fatehi et al., 2015, 2018). Especially in the U.S., where people who come from diverse cultural/ethnic backgrounds display different views toward collectivism and individualism despite living in a predominantly individualistic culture (Vandello and Cohen, 1999; Chiou, 2001). Although it has been inferred that those who maximize or satisfice would act differently across cultural contexts, the relationships between cultural dimensions and maximizing or satisficing decision styles has not been measured directly. Additionally, it remains unknown how an individual’s tendencies to maximize or satisfice may shape their personal views toward individualism. To fill these gaps, the current study investigates the relationship between maximizing and satisficing and individual cultural orientation in the U.S. using the Triandis and Singelis (1998) SINDCOL instrument. A second study is then conducted in Slovenia to see if the relationships operate differently across national contexts.

### Entrepreneurial decision-making

“Entrepreneurship, in its narrowest sense, involves capturing ideas, converting them into products and, or services and then building a venture to take the product to market” (Johnson, 2001, p. 138). Researchers have investigated numerous trait variables that have been linked to entrepreneurial intentions and behavior, finding that entrepreneurs are generally risk takers (Antoncic et al., 2018) who apply an individualistic approach when working with others (McGrath et al., 1992); they show resilience that allows them to surmount obstacles (Vizcaíno et al., 2021), and prefer to take action rather than be complacent (Lee et al., 2021), using their interpersonal skills to work with others to get things done (Clark, 2008). Although there are many traits associated with potential entrepreneurs, there is no archetypal entrepreneurial since the processes and skills vary greatly from one entrepreneurial venture to the next, and people may choose to pursue a career in entreprenership for a variety of reasons.

As a rapidly growing field, social entrepreneurship attempts to meet the needs of society through innovative solutions (Urban and Kujinga, 2017). The rise of social entrepreneurial education and important role that social entrepreneurs play in implementing social causes has become widely accepted (Stecker, 2014; Stoica et al., 2020), establishing a need to identify what may encourage individuals to pursue social change or meet societal needs by exploiting new opportunities. Social entrepreneurship has been defined as the combination of resources arranged to produce either new services, products, or organizations, with the intent of exploiting opportunities to accelerate social change, meet social needs, or increase social value (Kedmenec and Strašek, 2017).

Social entrepreneurship focuses on the “social” aspect of entrepreneurial activities, and therefore can be considered as a subcategory of entrepreneurship, with many overlapping activities (Tan et al., 2020). The main difference between social and traditional entrepreneurs lies with their intent to solve social problems or carry out a social mission (Zahra et al., 2008), and thus they tend to exhibit high levels of empathy and have a strong sense of moral obligation (Hockerts, 2017). Because the growth of entrepreneurship depends on the number and quality of entrepreneurs (Krueger, 2003), it follows that increasing the number of social entrepreneurs is necessary to expand the development of social entrepreneurial ventures. Perhaps looking at cultural differences in the way people approach entrepreneurial decisions may hold important clues for how we can identify and train individuals who are apt to find innovative solutions that solve social problems.

Researchers have long discussed the influence of cultural dimensions on personal choice. As an underlying system of values, culture shapes the way people think about and engage in behaviors (Mueller and Thomas, 2001). As such, culture has been seen as a motivating force for new venture creation, which serves to stimulate economic growth through new job creation. Based on the theory of social legitimation and moral approval, entrepreneurship rates are higher where social status elevates entrepreneurs as a desirable occupation (Etzioni, 1987). Among developed economies, there appears to be a positive relationship between individualistic cultures and entrepreneurial activity, as measured by new business starts according to the Global Entrepreneurship Monitor (Pinillos and Reyes, 2011). Fueling this entrepreneurial potential, it has been argued that individualistic cultures create more supportive environments that value pursuing personal goals through entrepreneurial means (Liñán et al., 2016). Along with the high need for achievement through the pursuit of personal rewards, having an internal locus of control and an innovative mindset may create the perfect recipe for those who desire to start new enterprises (Mueller and Thomas, 2001; Pinillos and Reyes, 2011).

Yet, an individualistic mindset may only benefit new business starts in economies that have a level of economic development conducive to facilitating personal entrepreneurial endeavors and some have argued that collective cultures may be more favorable to entrepreneurs in less developed economies, where there can be greater cooperative support for such an undertaking (Pinillos and Reyes, 2011; Zeffane, 2014). At the national level, the connection between individual – collective cultures and entrepreneurship has seen varied results (Hunt and Levie, 2002; Pinillos and Reyes, 2011). The mixed results have been attributed to an oversimplification of the way in which national culture may influence entrepreneurial decisions at the personal level (Wennberg et al., 2013). Similarly, at the national level, there appears to be no clear relationship between the individualism - collectivism cultural dimensions and social entrepreneurial intentions (Kedmenec and Strašek, 2017). Intriguingly, there have only been a few studies measuring the relationship between cultural perspectives and entrepreneurial intentions at the individual level, and these have been done outside the U.S. in Spain (Liñán et al., 2011), Finland (Rantanen and Toikko, 2017), United Arab Emirates (Zeffane, 2014), and Pakistan (Farrukh et al., 2019). There have not been any studies exploring how personal cultural views may shape social entrepreneurial intentions. Therefore, there is a need to measure the relationship between cultural orientation and entrepreneurial intentions at the individual level in the U.S. and in Slovenia to see if personal cultural views may shape individuals’ propensity to start new business ventures, and the type of ventures they form.

## Model construction and theoretical hypothesis

### Maximizing or satisficing and cultural orientation

Triandis and Gelfand (1998) argue that, within any culture, individualism and collectivism can exist simultaneously within any individual and may be different than the prevailing cultural norms. Thus, measuring culture at an individual level may offer more depth to understanding cultural perspectives that exists within individuals who reside within cultures that may take on prevailing norms, and how those perspectives may be linked to personal trait variables. Because maximizing is associated with wanting to be the best through individual choices (Schwartz et al., 2002) and controlling the decision process (Sparks et al., 2012), it may lend toward a mindset that views oneself more independently rather than connected with others. Maximizers view themselves comparatively to others, trying to outdo those who are doing better than them (Schwartz et al., 2002; Weaver et al., 2015). These social comparisons push them to apply more effort into achieving their goals and attaining superior outcomes (Chan, 2021). Others have found that they are more achievement oriented, focused on outcomes rather than the process (Hsieh and Yalch, 2020), and will pursue high value but effort consuming opportunities (Luan et al., 2018). In a study of self vs. other decisions, maximizers sought out the best options for themselves and others, whereas satisficers prefer the best options for others but did not put forth the same effort for themselves (Luan et al., 2018), suggesting that they may have greater concern for group goals over their own. Similarly, maximizers are more concerned with how their outcomes compare to others than their objective results (Weaver et al., 2015). Overall, it appears that maximizers relate to others through a competitive lens, basing their search strategies around external validation (Iyengar et al., 2006; Parker et al., 2007; Luan et al., 2018) social comparisons (Schwartz et al., 2002; Polman, 2010), and being the best among their peers (Weaver et al., 2015; Chan, 2021). For this reason, they are more likely to spend greater effort to search for the best in public vs. private domains (Luan et al., 2018), where the results of their decisions become part of their social status.

As defined by Triandis (2018, p. 2), Individualism is “a social pattern that consists of loosely linked individuals who view themselves as independent of collectives; are primarily motivated by their own preferences, needs, rights, and the contracts they have established with others; give priority to their personal goals over the goals of others; and emphasize rational analyses of the advantages and disadvantages to associating with others.” Because maximizers are more concerned with status and view themselves on a comparative basis with others rather than seeing themselves as part of a group of equals, they may have a more individualistic cultural perspective. For example, maximizers are more concerned with status in consumer decisions (Brannon and Soltwisch, 2017) and attempt to outdo their peers by searching for better job opportunities (Iyengar et al., 2006). As defined by Waterman (1984), normative individualism relies on freedom of choice and personal responsibility, respecting the integrity of others, and living up to one’s potential. It has been associated with the values of demonstrating one’s competence to others through achievement and displaying successes through social recognitions (Nelson and Shavitt, 2002). Underlying the maximizing trait is the assumption that making good decisions is a means for achieving superior results, which may further one’s goals toward achieving their objectives. Maximizers are achievement oriented (Peng et al., 2018) and expect better outcomes due to their intensive search strategies (Iyengar et al., 2006; Benjamin et al., 2014). Based on aspects of maximizing related to self-other decisions, their use of social comparisons as a means for status and having a high need for achievement through effortful decision search and comparative analysis, it is predicted that maximizers will be more individualistic in their cultural perspective.

*H1*: Those who maximize their decisions will have a more individualistic cultural view.

### Cultural orientation and entrepreneurial intentions

Because of its relation to autonomy and independence as defined by the Hofstede’s (1980) model, individualism has been associated with entrepreneurial intentions in a variety of settings (Mitchell et al., 2000; Liñán and Chen, 2009; Siu and Lo, 2013; Liñán et al., 2016). For various reasons, individualism fits the profile of an entrepreneur. In individualistic cultures, people display a high need for achievement and are encouraged to pursue individual goals over group goals (Pinillos and Reyes, 2011). They foster innovation while applying an internal locus of control (Mueller and Thomas, 2001). By awarding social status to those who exhibit new discoveries, individualism has been connected to long-term economic growth and innovativeness (Gorodnichenko and Roland, 2012). It has been argued that entrepreneurial activity is more valued and encouraged in individualistic cultures through a more supportive environment (Liñán et al., 2011). For these reasons, there has been some support for the relationship between individualistic cultures and entrepreneurial behavior (McGrath et al., 1992; Mueller and Thomas, 2001; Wennekers et al., 2002; Pinillos and Reyes, 2011; Liñán et al., 2016).

Yet, the national level data tends to indicate that this relationship may only hold in highly developed economies. Based on Global Entrepreneurship Monitor data, a study of 52 countries found that in nations with low or medium economic development, individualism did not have any effect on entrepreneurial activity (Pinillos and Reyes, 2011). The authors conclude that the needs of self-fulfillment and personal achievement may only be satisfied when economic conditions can support an individual’s personal efforts toward entrepreneurial endeavors. Others have found that at the national level, culture explains only a small portion of the variance in individual attitudes toward entrepreneurship and entrepreneurial activity (Hunt and Levie, 2002). Because these studies assume that everyone within a nation takes on the predominant cultural perspective, it has been argued that they may be missing individual nuances in cultural views that shape personal attitudes toward entrepreneurship that extend to new venture decisions (Rantanen and Järveläinen, 2016).

Surprisingly, there have only been a few studies investigating how personal cultural views shape entrepreneurial intentions. In a comparison of various regions of Spain, Liñán et al. (2016) identified that when a person is more individualistic than the prevailing cultural norms in the region, they tend to show greater intentions toward entrepreneurship (Liñán et al., 2016). In Finland, it was found that both individualism and collectivism was associated with greater entrepreneurial intentions (Rantanen and Järveläinen, 2016). The authors note that social bonds among those in collective cultures can encourage entrepreneurship, especially in lower economic development regions. Research in Pakistan has found that individualistic personal cultural views were strongly associated with new venture intentions when people perceive that entrepreneurship is within their reach and when they have a positive attitude toward entrepreneurship (Muhammad et al., 2019). Interestingly, similar to the study in Finland, Pakistani students who were more collectivist in their views, were more interested in entrepreneurship when cultural norms supported it. This suggests that the level of societal support for new business ventures can be an influential factor, especially for those who live in collective cultures.

Yet, in the U.S. it remains relatively unknown how personal cultural views affect entrepreneurial intentions. As a nation, the U.S. is generally supportive of individual achievements through entrepreneurial means and has developed an economic model based on growth through new business innovation. National data supports this assertion as it is ranked 12th globally based on the Global Entrepreneurship Monitor (GEM) data for favorable entrepreneurial environments. Pinillos and Reyes (2011) similarly found a strong correlation between individualistic national culture and entrepreneurial activity in the U.S. according to GEM data, noting that the relationship between individual culture and entrepreneurship applied best to nations that have a high enough level of economic development to support individual pursuits toward achieving entrepreneurial goals. According to Hofstede’s research, the U.S. has an individualistic culture that promotes individuality and decision-making autonomy as a means for achieving success.

However, others have argued for a more nuanced approach to measuring cultural differences among citizens in the U.S. to account for the wide variety of cultural backgrounds and perspectives based on the country’s history of bringing together diverse racial and ethnic populations (Triandis and Gelfand, 1998). For example, these personal differences in individualistic perspective become apparent when comparing states within the U.S. that have different views toward individualism and collectivism (Vandello and Cohen, 1999). Additionally, there may be generational differences as levels of individualism are rising among young adults who are less engaged in community life as those of past generations (Nezlek and Humphrey, 2021). However, for entrepreneurs it appears that individualism may be beneficial to well-being. For example, Atalay and Tanova (2021) found that entrepreneurs experience greater well-being in individualistic cultures due to having more autonomy to make decisions in a way that produces desirable results. Entrepreneurs are driven by independence and the freedom to make their own decisions (Douglas and Shepherd, 2002; Shepherd et al., 2015). In cultures that promote this independence, such as the U.S., it is conceivable that those who have a more individualistic cultural perspective may see entrepreneurship as a desirable career choice to achieve personal goals. Therefore, it is predicted that an individualistic cultural perspective among individuals in the U.S. will be positively related to entrepreneurial intentions.

*H2*: Individualism will be positively related to entrepreneurial intentions.

Through the discovery and exploitation of opportunities, social entrepreneurs find their purpose in serving society rather than their own interest (Mair and Noboa, 2003, 2006). Although there has been little research on how cultural orientation may impact social entrepreneurial intentions directly, a study comparing the U.S. with China found that attitude toward entrepreneurship was a more important predictor of social entrepreneurial intentions in the U.S. than in China (Yang et al., 2015). The authors attribute this to the individualistic nature of the culture in the U.S., where people are more likely to be motivated by their own interests and attitudes. In line with other studies in collective societies, they found that societal norms toward entrepreneurship were more influential in predicting entrepreneurial intentions in China (compared to the U.S.) based on the role that society and significant others may play in supporting and encouraging social entrepreneurship to serve others. At the personal level, in societies that support personal achievements over group outcomes, it is possible that individualistic values would be less aligned with the collective goals of social enterprises. Therefore, it is predicted that individualism will be negatively related to social entrepreneurial intentions.

*H3*: Individualism will be negatively related to social entrepreneurial intentions.

### Maximizing and entrepreneurial intentions

According to the Theory of Planned Behavior (Ajzen, 1991), entrepreneurial intentions are indicative of the effort an individual is prepared to carry out to start a new business venture, which has been a reliable predictor of entrepreneurial behaviors (Van Gelderen et al., 2008; Wu, 2009). For example, studies have found that personality traits such as need for achievement (Tong et al., 2011), self-efficacy (Zhao et al., 2005), internality of control (Tentama and Abdussalam, 2020), tolerance for risk (Segal et al., 2005) and conscientiousness (Engle et al., 2010) are related to increased intentions to start new ventures. Because maximizers are persistently comparing options to find better alternatives (Schwartz et al., 2002), it makes sense that recent studies have begun to investigate the role of these decision styles in the areas of innovation and entrepreneurship.

Among entrepreneurs, those who maximize their decision efforts tend to build more entrepreneurial and market-oriented firms that can better meet changing market demands, resulting in superior financial performance (Soltwisch, 2021). Given that maximizers persistently seek out better alternatives (Schwartz et al., 2002), prefer to control the decision-making process (Sparks et al., 2012), are more confident in their abilities to lead, and will put forth additional effort to achieve superior results as executives (Lai, 2010), they share many of the same traits that exemplify the entrepreneurial profile. Maximizers tend to feel more personally responsible for the outcomes of their decisions (Schwartz et al., 2002). In societies that view success as the result of personal achievements through good decision-making, such as the U.S., maximizers tend to be extra critical of themselves because they compare their outcomes with those of others (Roets et al., 2012). In individualistic cultures, where personal achievements through independent efforts are revered, and entrepreneurship is promoted as a path for obtaining superior personal and financial rewards, maximizers may be more likely to view entrepreneurship as a means to achieve greater relative success.

Recently, it has been found that maximizers in the U.S. have a more innovative mindset and are more entrepreneurially orientated, which makes them more likely to see entrepreneurship as a viable career path (Soltwisch et al., 2022). It makes sense that entrepreneurship may be a viable means for achieving better results for those who maximize, especially in the U.S., where the economic environment may favor those who take steps to create a better future for themselves. Therefore, in line with existing work, it is predicted that maximizing will increase individual’s intentions to start new business ventures in the U.S.

*H4*: Maximizing will be positively related to entrepreneurial intentions in the U.S.

Beyond optimizing outcomes for themselves, maximizers also attempt to find better solutions for others and will encourage others to maximize their decision efforts (Luan et al., 2018). In doing this, they will encourage those around them to pursue highly valued goals that require significant effort. The goals of social entrepreneurs often require considerable effort, and it is important for entrepreneurs to build support for others to help reach the venture’s goals. Maximizers are more innovative and identify additional opportunities for new businesses in their environment (Soltwisch et al., 2022). They persistently seek out additional information and options to find better solutions. It is possible that they would apply this innovative mindset to identifying social problems that may create entrepreneurial opportunities. Maximizers are more likely to view ethically questionable situations as being immoral based on their absolutist ideology (Soltwisch et al., 2020). This principles-based view considers that ethical standards should be applied uniformly across all people and may be a basis for addressing the concerns of underserved populations. Because maximizers tend to be more attuned to see ethical issues as immoral and may identify more opportunities to solve those problems, the current study aims to investigate whether maximizing may increase an individual’s intentions to start socially oriented businesses with a mission to serve others. Because the relationship between maximizing and social entrepreneurial intentions has not been previously tested, this will provide a first look into how one’s search for the best may relate to solving social problems through entrepreneurial means.

*H5*: Maximizing will be positively related to social entrepreneurial intentions in the U.S.

**Figure 1:**
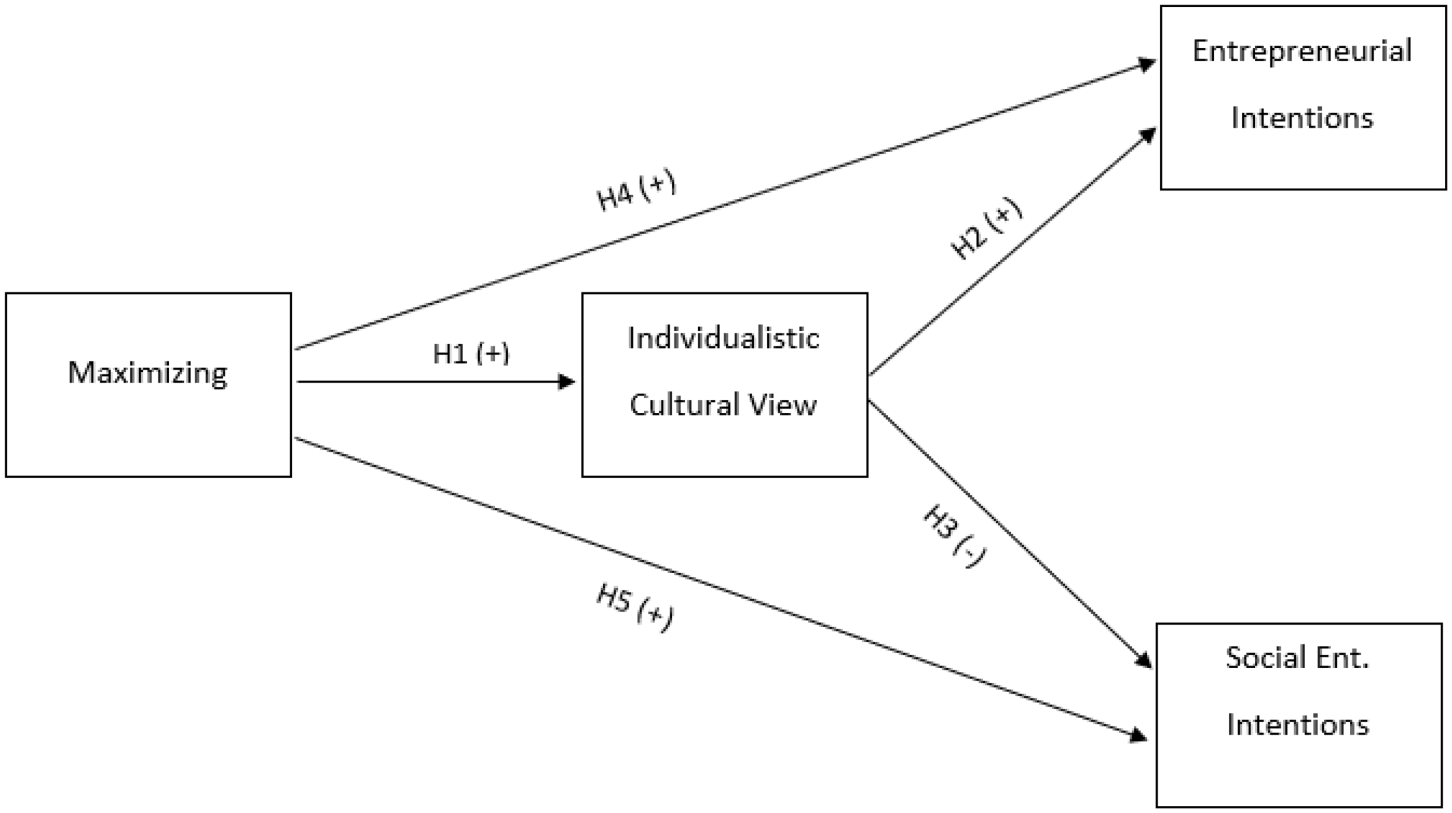
Research model.

To recap the discussion thus far, it was hypothesized that maximizers will have a more individualistic view. Further it was posited that those who are more individualistic will have greater entrepreneurial intentions but lower intentions to start social enterprises in the U.S. Thus, taken together with hypotheses H4c and H5C, individualistic cultural views may mediate the relationships between maximizing and the dependent variables of entrepreneurial intentions and social entrepreneurial intentions. Therefore, the proposed research model can be found in Figure 1 below.

### Methods and results

Two hundred and five students taking upper-level business courses at a university in the Western U.S. completed a survey measuring the focal variables in the study (45% female, mean age = 22.22). Participants completed a questionnaire using Qualtrics survey software. In exchange for their participation, participants were offered nominal extra credit. Among those who completed the survey, 188 students provided valid data. Seventeen respondents were removed due to missing data on either the independent or dependent variables. To measure culture, 12 items measuring individualism were used from the SINDCOL instrument (Triandis and Singelis, 1998, p. 42–47; Cronbach’s α = 0.67). Although the reliability was low, it was similar to that found by Singelis (1994), with Cronbach’s α =0.69 in the first study and.70 in the second. Questions measure various aspects of individualism such as, “Would you say that most of the time you do “your own thing” paying no attention to whether or not it fits customs and “proper” behavior? Respondents are asked to rate their behavior on a 10-point scale, with a 10 as most likely.

Entrepreneurial intentions were measured using Liñán and Chen’s (2009) six-item scale (Cronbach’s α = 0.96). Examples of items include: “I am ready to do anything to be an entrepreneur”; and “I have the firm intention to start a firm someday.” Hockerts (2017) Social Entrepreneurial Intention Scale was used to measure intentions to start ventures with social missions (Cronbach’s α = 0.85). The scale asks questions such as, “I expect that at some point in the future I will be involved in launching an organization that aims to solve social problems”; and “I have a preliminary idea for a social enterprise on which I plan to act in the future.”

To measure the independent variable (maximizing), participants completed the nine-item Maximizing Tendency Scale (MTS; Highhouse et al., 2008; Cronbach’s α = 0.97). Some examples of the Maximizing Tendency Scale items include: “My decisions are well thought through”; “I never settle”; and “I am a maximizer.” Finally, students completed demographic variables and described their ethnic background by answering the question, “what is the most important source of your ethnic background”? Participants selected from 17 ethnic backgrounds identified by Triandis and Singelis (1998) representing regions from around the world. The largest three ethnic backgrounds represented in the U.S. sample were Western European (51%), Northern European (25%), and Mexico (9%). Table 1 below shows the correlations among focal variables in the study.

**Table 1:** Correlations among variables.

		Means	Range	St. Dev.	1	2	3	4	5	6	7
1	Maximizing	5.37	1–7	0.86	1.						
2	Individualism	5.48	2–8.6	1.08	0.23**	1.					
3	Ent Intentions	4.14	1–7	1.74	0.29**	0.26**	1.				
4	Soc Ent. Intentions	4.29	2–7	0.89	0.28**	0.11	0.38**	1.			
5	Age	22.22	18–72	4.9	−0.07	0.06	−0.04	0.04	1.		
6	Gender	1.46	1–2	0.5	0.02	−0.17*	−0.23**	−0.16*	−0.15*	1.	
7	Ethnic Background	5.74	1–7	0	−0.03	0.0	−0.11	−0.13	−0.01	0.08	1.

*N* = 188. Gender (1 = male, 2 = female).

** *p* < 0.01.

To recap the predictions, it was posited that maximizers will have a more individualistic view. Further it was expected that individualism will have a positive relationship to entrepreneurial intentions and a negative relationship with social entrepreneurial intentions. Therefore, taken together, an individualistic cultural view may mediate the relationships between maximization and the dependent variables entrepreneurial intentions and social entrepreneurial intentions. The control variables of age, gender, and ethnic background were included in all regressions to account for variations in cultural orientation and entrepreneurial intentions due to gender, age, or ethnicity. In the current sample, men were significantly more likely to be individualist and showed significantly higher entrepreneurial intentions.

To test the relationship between maximizing and individualistic views, individualism scores were regressed on participants’ maximization scores. As expected in H1, results indicate that those who maximize are significantly more individualistic in their cultural view [b = 3.56, *t*(184) = 3.30, *p* < 0.01]. The relationships between individualism and the dependent variables entrepreneurial intentions and social entrepreneurial intentions (H2, H3) was tested using multiple regression analysis in SPSS, finding that individualism was significantly related to higher entrepreneurial intentions [b = 0.03, *t*(184) = 3.26, *p* < 0.01], but unrelated to social entrepreneurial intentions [b = 0.006, *t*(184) = 1.24, *p* = 0.22]. Thus, hypothesis H2b was supported, while H3 was not. Finally, the dependent variables of entrepreneurial intentions and social entrepreneurial intentions were regressed on maximization scores to test hypotheses H4c and H5, finding that maximization significantly increased entrepreneurial intentions [b = 0.61, *t*(184) = 4.42, *p* < 0.01] and social entrepreneurial intentions [b = 0.3, *t*(184) = 4.42, *p* < 0.01], supporting hypothesis H4 and H5. See Table 2 displaying regression results for the independent variables maximizing and individualism on the dependent variables.

**Table 2:** Regression results for maximization and individualism.

Independent Variables	Maximizing							Individualism			
Dependent variables	Individualism		Entrep Int		Soc Entrep Int			Entrep Int		Soc Entrep Int	
	β	*t*	β	*t*	β	*t*		β	*t*	β	*t*
Age	0.0.01	0.78	−0.02	−0.8	0.01	0.6	Age	−0.03	−1.22	0.01	0.24
Gender	−0.37	−2.37	−0.84**	−3.48	−0.28**	−2.17	Gender	−0.69**	−2.75	−0.24	−1.79
Ethnicity	0.01	0.27	−0.08	−1.24	−0.05	−1.58	Ethnicity	−0.09	−1.39	−0.06	−1.66
Maximization	0.29**	3.3	0.61**	4.42	0.3**	4.18	Individualism	0.37**	3.26	0.07	1.24
Model R^2^	0.09		0.16		0.13		Model R^2^	0.12		0.05	
Adjusted R^2^	0.07		0.14		0.11		Adjusted R^2^	0.1		0.03	
Model F	4.27**		8.57**		6.47**		Model F	6.19**		2.33	

*N* = 188. Gender (1 = male, 2 = female).

** *p* < 0.01.

To test the mediation effects of individualism on the relationship between maximizing and entrepreneurial intentions and social entrepreneurial intentions, model 4 of the bootstrapping process described by Hayes and Preacher (2014) was used with 5,000 samples. The control variables of age, gender, and ethnic background were included as covariates (Figure 1). For the maximizing - individualism – entrepreneurial intentions mediation model, the first path (H1) showed that maximizing was significantly related to individualism [b = 0.29, *t*(180) = 3.29, *p* < 0.01]. The second path (H2) found that individualism was significantly related to entrepreneurial intentions [b = 0.26, *t*(180) = 2.38, *p* < 0.05]. And the last path (H4) showed that maximizing was significantly related to entrepreneurial intentions [b = 0.52, *t*(180) = 3.77, *p* < 0.01]. The bootstrapping results indicate that individualism fully mediated the path between maximization and entrepreneurial intentions (b = 0.61, CI_95%_ exclusive of 0 [0.009, 0.182]).

**Figure 2:**
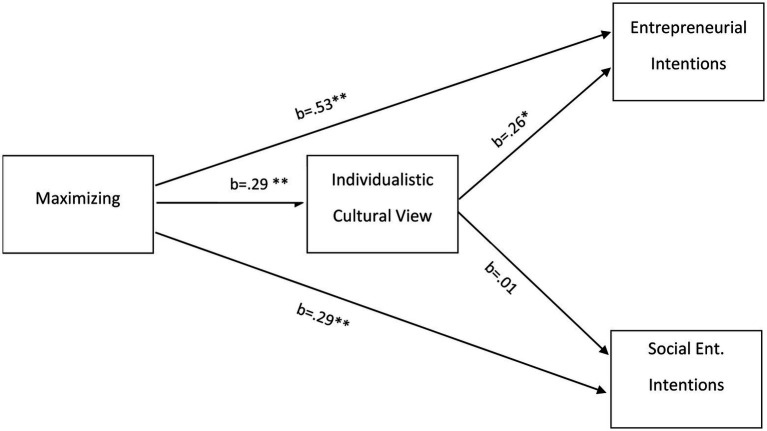
Mediation model. *p < .05; **p < .01.

For the second model testing the maximizing – individualism – social entrepreneurial intentions relationship, the first path found that maximizing was significantly related to individualism [b = 0.29, *t*(180) = 3.29, *p* < 0.01]. The second path was not significant [b = 0.01, *t*(180) = 0.30, *p* = 0.76], indicating that individualism was not related to social entrepreneurial intentions. Thus, the mediation of individualism on the relationship between maximizing and social entrepreneurial intentions was not supported (b = 0.30, CI_95_ [−0.03, 0.05]). See Figure 2 displaying the mediation results.

## Study 2

A second study was conducted in Slovenia to test how the relationships would operate in a more collectivistic national culture. With the goals of expanding generalizability and identifying boundary effects for the proposed relationships, this study serves to answer a call for more cross-cultural comparative research on the antecedents of entrepreneurial intentions (Antoncic and Hisrich, 2001; Liñán and Chen, 2009). In 2020, Slovenia was ranked 12th on the economic complexity index, just behind the 9th ranked U.S. according to EOC data which compiles a variety of data points to measure the productive capabilities of large economies (OEC, 2020). Thus, similar to the U.S., the Slovenian economy can support a high level of economic activity. Slovenia has successfully transitioned from a socialist country to a market-based economy (Hisrich et al., 2003) and the government has actively supported entrepreneurship as a means for promoting economic development. Yet, the transition has been slow to impact new business starts, with a total entrepreneurial activity (TEA) score of around 6.66, according to the most recent Global Entrepreneurship Monitor (GEM) data (GEM, 2020/2021 report). In comparison, the U.S. has a total entrepreneurial activity (TEA) score of 23.06 (GEM, 2020/2021 report).

It is true that the U.S. and Slovenian economies share some commonalities despite their differences in size, however, the distinct economic and cultural history of Slovenia may shape the way people perceive entrepreneurship. Historically, Slovenians have adopted a predominantly collective culture, and economic decisions were based on mutual benefit rather than personal gains. For example, Musek (2004) showed that people in Slovenia ranked socially based values as their top priority. Slovenian’s generally carry strong bonds with their family, have traditional values, and prefer to remain rooted near their homes, often living with several generations in one household (Penger et al., 2015). Based on Hofstede’s data, Slovenia has an individualism score of 27, suggesting a more collectivist society. In comparison, the U.S. marks a 91 on individualism (Hofstede Insights Organisational Culture Consulting, 2022).

Because Slovenia has an advanced level of economic development, yet a unique historical and cultural perspective relative to the U.S., it is an ideal place to compare students’ perceptions of entrepreneurial opportunities based on differences in their cultural orientations and decision styles. Additionally, the second study investigates further distinctions in cultural dimensions that may be related to maximizing and satisficing. In study 2 cultural orientation is measured using Triandis and Gelfand’s (1998) measurement of vertical and horizontal individualism and collectivism, which identifies two individualism dimensions (vertical and horizontal) and two collectivism dimensions (vertical and horizontal). Building on Triandis’ (1995) recognition that a distinction between the vertical and horizontal I and C also needs to be made, the Study of Triandis and Gelfand (1998) was pivotal in introducing the idea that being just a little individualist (I.) and a little collectivist (C.) is not enough for a person from a certain national culture. Rather, there is another level of analysis – personal, individual, identity crucial, and unique. This is the horizontal/vertical (H/V) aspect of the individual cultural differences.

These distinctions are defined by Singelis et al. (1995) as (1) horizontal individualism is a cultural pattern where the self is autonomous and independent from others, but yet equal in status to them – perceived as same; (2) Vertical individualism is a cultural pattern where the self is autonomous, but different from others. Inequality and competition are the expectation in this cultural orientation; (3) Horizonal collectivism (H-C), is a cultural pattern where the self is merged with the members of an in-group and personal identity is perceived as part of the identity of an in-group; (4) And vertical collectivism (V-C) is a cultural pattern where the self is still a part of the in-group, but not the same and not equal to the other selves.

Measuring vertical and horizontal dimensions of individualism may reveal whether maximizing is more related to status distinctions (vertical individualism) or the desire to make decisions autonomously (horizontal individualism). The horizontal and vertical collectivism dimensions will allow for the distinction between seeing oneself as part of a group and identifying with the group’s goals (horizontal collectivism) or being part of a group but not prioritizing group goals (vertical collectivism). The independent variable (maximizing) and dependent variables (entrepreneurial intentions, social entrepreneurial intentions) remain the same as in study 1 to cross-validate the findings in a different national context.

### Sample and measures

Students attending the University of Ljubljana and the University of Primorska in Slovenia completed a survey measuring the studied variables. Students were sent a link to complete the survey using the Qualtrics survey software. Students were asked to select the country where they were born or spent the greatest part of their formative years 1–10. Out of 152 students who completed the questionnaires, 107 indicated they were from Slovenia or spent their formative years there, and thus were included in the analysis. Triandis and Gelfand (1998) developed a 16-item scale measuring 4 items for each cultural orientation: vertical individualism (Cronbach’s α = 0.88 in current study), horizontal individualism (Cronbach’s α = 0.82 in current study), vertical collectivism (Cronbach’s α = 0.81 in current study), and horizontal collectivism (Cronbach’s α = 0.81 in current study). Consistent with study 1, the Maximizing Tendency Scale (MTS; Highhouse et al., 2008; Cronbach’s α = 0.79), Entrepreneurial Intentions Scale (Liñán and Chen, 2009; Cronbach’s α = 0.97), and Social Entrepreneurial Intention Scale (Hockerts, 2017; Cronbach’s α = 0.91) were used to measure maximizing, entrepreneurial intentions, and social entrepreneurial intentions, respectively.

### Results

Multiple regression analysis was used to test the relationships proposed in study 1; however, this time the relationships between maximizing and two individualism and two collectivism dimensions of culture were considered. The control variables of age and gender were included in the regressions to account for any variations in the entrepreneurial intentions that may be related to these factors. To test the relationship between maximizing and vertical and horizontal individualism, respondents vertical individualism scores were regressed on their maximization scores [b = 0.83, *t*(104) = 5.8, *p* < 0.01], suggesting that maximizing was significantly related to higher vertical individualism. Similarly, maximizing was significantly related to higher horizontal individualism scores [b = 0.57, *t*(104) = 6.0, *p* < 0.01]. Thus, Slovenian students who maximize were more individualistic on both the vertical and horizontal dimensions, offering further support for H1. Next, for contrast, the relationships between maximization and vertical and horizontal collectivism are tested to see if maximizing was unrelated to collectivism. Respondents vertical and horizontal collectivism scores were regressed on their maximization scores, resulting in a slightly negative but insignificant relationship between maximizing and vertical collectivism [b = −0.02, *t*(104) = −0.13, *p* = 0.89] and horizontal collectivism [b = −0.10, *t*(104) = −1.0, *p* = 0.31]. Thus, among Slovenian students, maximizing appears to be positively related to both dimensions of individualism and unrelated to vertical and horizontal collectivism. See Table 3 for descriptive statistics and Table 4 for regression results of individualism and collectivism dimensions based on maximization scores.

**Table 3:** Study 2 descriptive statistics.

Descriptive statistics				
	Minimum	Maximum	Mean	Std. Deviation
Vertical Individualism	1.00	7.00	4.02	1.32
Horizontal Individualism	3.25	7.00	5.71	0.87
Vertical Collectivism	2.50	7.00	5.07	1.03
Horizontal Collectivism	2.00	7.00	5.54	0.82
Entrepreneurial Intentions	1.00	7.00	4.08	1.64
Age	20.00	50.00	23.61	5.35
Gender	1.00	2.00	1.70	0.46
Country (Slovenia)	1.00	1.00	1.00	0.00

Gender (1 = male, 2 = female) 70% of respondents were female

**Table 4:** Regression results for vertical and horizontal individualism and collectivism.

	Vertical individualism		Horizontal individualism		Vertical collectivism		Horizontal collectivism	
	β	*t*	β	*t*	β	*t*	β	*t*
Age	−0.05*	−2.43	−0.05**	−3.58	0	0.01	0.02	0.247
Gender	−0.54*	−2.24	0.18	1.14	−0.51*	−2.31	−0.18	0.31
Maximization	0.83**	5.8	0.57**	6	−0.02	−0.13	−0.11	0.31
Model R^2^	0.29		0.29		0.05		0.03	
Adjusted R^2^	0.27		0.27		0.02		0.01	
Model F	14.18**		14.1**		1.83		1.04	

*N* = 107.

* *p* < 0.05;

** *p* < 0.01.

**Figure 3:**
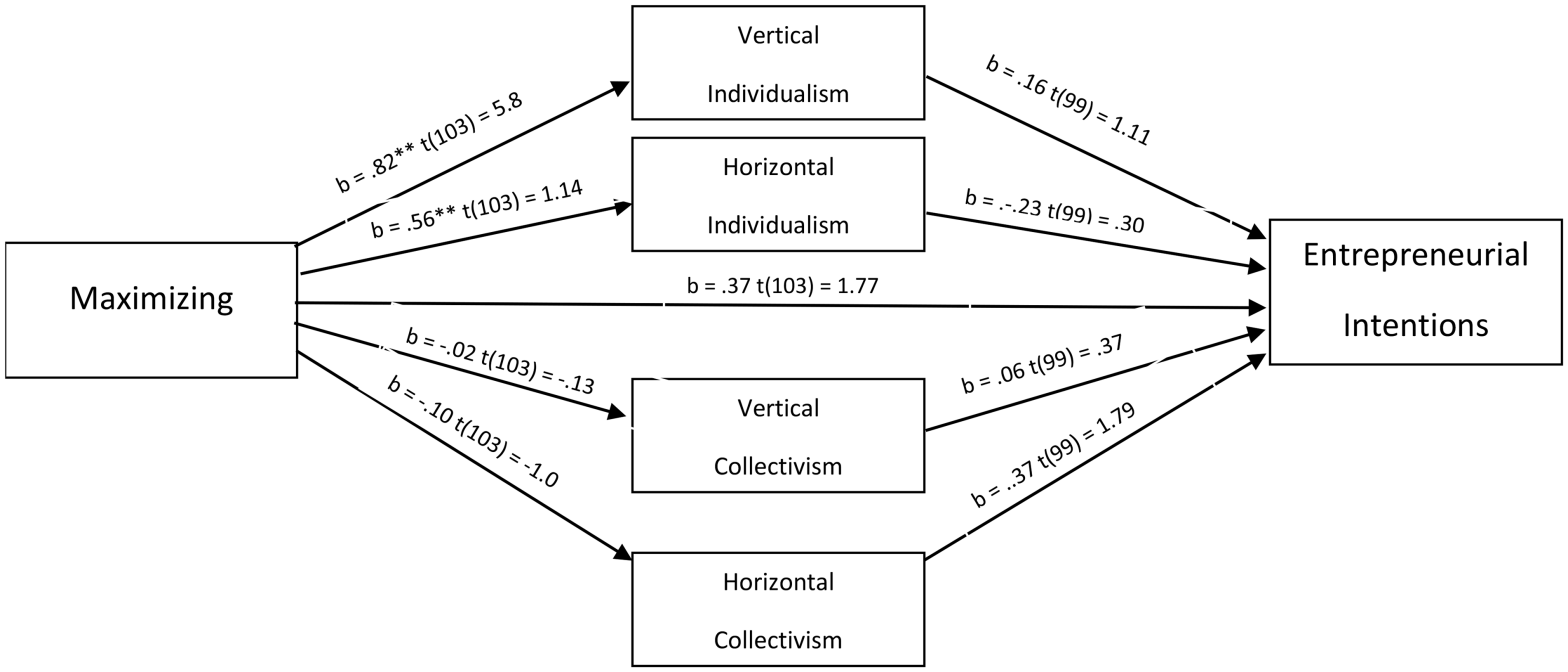
Mediation results of study 2.

Next, the relationships between maximizing and entrepreneurial and social entrepreneurial intentions were tested using multiple regression analysis, with the controls of age and gender included. Results suggest that, among Slovenian students, maximizers have greater entrepreneurial intentions [b = 0.37, *t*(104) = 1.78, *p* < 0.1, *p* = 0.07], however, this time at a marginally significant level. Interestingly, maximizing did not increase social entrepreneurial intentions among Slovenian students [b = −0.19, *t*(104) = −0.14, *p* = 0.18]. To test whether the dimensions of individualism or collectivism may mediate the relationship between entrepreneurial intentions and maximizing, similar to what was found in study 1, parallel mediation was employed using model 4 of the bootstrapping process described by Hayes and Preacher (2014) with 5,000 samples. This time there were no interaction effects for the cultural dimensions on the positive relationship between maximizing and entrepreneurial intentions (b = −0.04, CI_95%_ [−0.34, 0.33]). Despite the limited sample size in study 2, Cohen’s D indicated large effect sizes for the relationships with vertical (d = 1.14, *r* = 0.49) and horizontal individualism (d = 1.18, *r* = 0.50), suggesting adequate explanatory power. The mediation results of study two can be found in Figure 3.

## Discussion

This study takes a first step in exploring how our decisional preferences of maximizing or satisficing relate to our cultural perspective and our tendencies to launch new business ventures. Specifically, it was found that among students in the U.S., those who maximize have a more individualistic cultural perspective, which tends to increase their intentions to become entrepreneurs. With a growing interest in social entrepreneurship worldwide (Zahra et al., 2014), the study also explores how our decision styles and cultural orientation may impact individual’s intentions to start social enterprises. It appears that we can now add maximizing to the list of important factors that may encourage individuals to pursue entrepreneurial opportunities with a social mission. This makes sense given maximizers’ constant search for better alternatives combined with their goals to maximize outcomes for themselves and others (Luan et al., 2018). As philanthropic and government funding for non-profit organizations becomes less sustainable in the U.S., social entrepreneurship has become an increasingly important means for solving social issues (Stecker, 2014) while creating new job opportunities (Rey-Martí et al., 2016).

It appears that among high maximizing individuals in the U.S., those who apply a more individualistic cultural orientation are more likely to seek out traditional entrepreneurial ventures, while those who are less individualistic are more inclined to start social enterprises. This distinction may be attributed to different entrepreneurial objectives based the way they view their role in society. Individualist view themselves autonomously, and independent from the group; prioritizing personal goals over those of the group, and viewing behaviors on a transactional basis (i.e., in exchange for payments; Triandis, 2001). For these reasons, it follows that those who apply an individualistic view may seek out traditional entrepreneurial opportunities over social ventures based on their personal incentives and the way they view social problems – perhaps seeing them as issues that are not best solved through entrepreneurial means.

By comparing students in the U.S. with students in Slovenia, it is apparent that the normative environment within national boarders plays an important role in shaping the way individual’s may consider entrepreneurial opportunities and the type of ventures based on their personal cultural perspectives. Similar to students in the U.S., Slovenian students who maximize their decisions were far more individualistic than those who were more satisficing in their choices. This result extended to both vertical and horizontal dimensions of individualism, offering additional support for the relationship between maximizing and individualistic cultural views in an international context. Interestingly, it appears that maximizing is related to status distinctions through comparative analysis (vertical individualism) and a desire for autonomy in making decision independently (horizontal individualism). Because maximizing may be an inheritable trait (Saad et al., 2020), this outlook of persistently searching for better alternatives may shape the way individuals interact with others throughout their life, altering their cultural views.

It is not surprising that achievement orientation through a maximizing decision style may be ubiquitous across national boundaries. However, when it comes to pursuing those lofty ambitions through entrepreneurship, it appears that the prevailing cultural and economic environment may play a role in the way individual’s view enterprising opportunities. Similar to the findings in study 1, Slovenian students who maximized had greater intentions to start entrepreneurial ventures; however, the relationship was only marginally significant, suggesting that maximizing students in Slovenian may not be as likely to pursue entrepreneurship as those in the U.S. It is possible that they consider more traditional career paths to meet their high desires for achievement. Interestingly, maximizers in Slovenia did not have greater social entrepreneurial intentions. Perhaps, pursuing new ventures is not the most viable means to tackle social problems based on Slovenia’s historical importance of the public sector handling social issues. It is also possible that there is limited funding for such undertakings at the individual level. Development of Slovenian social entrepreneurship is governed and monitored primarily by adopted Act on Social Entrepreneurship in 2011 (Tomaževič and Aristovnik, 2018). In October 2018, 259 organizations were officially registered as social enterprises in the register, fulfilling all required law criteria. Social innovation in Slovenia is still in its early stages and remains largely underdeveloped without the proper supporting environment for social innovators (Tomaževič and Aristovnik, 2018).

Past work on maximizing or satisficing has found that national context may reduce some of the negative psychological outcomes associated with preferences for finding the best through maximizing. However, based on the broad social, cultural, and economic differences between countries and regions of the world, this study answers the call for a more nuanced look at the relationship between decision styles and cultural perspectives (Oishi et al., 2014; Schwartz, 2016). As the first study to directly measure individuals’ cultural outlook based on their decision style (maximizing or satisficing), the results suggest some exciting applications to current theory and practice. It appears that in both the U.S. and Slovenia, maximizers are more inclined to apply an individualistic cultural view. Thus, they may prefer to work on their own terms, pursue individual goals and recognitions over collective ones, and may be reluctant to accept prevailing norms or submit to authority. These independently minded individuals may be well suited for innovative roles that provide a high level of autonomy over the decision-making process. For example, a new product lead, organizational manager, corporate entrepreneur or independent business owner may be positions that fit their personal dispositions toward searching extensively for better options.

Interestingly, individualism does not appear to be related to social entrepreneurial intentions in either the U.S. or Slovenian samples. It is possible that for those who seek status through individual achievements, social purposes may be less compelling than other reasons to start new ventures, such as financial gains or desires to be their own boss. This would align with individualistic characteristics of viewing oneself autonomously and independent from the group, prioritizing personal goals over those of others, and acting on a transactional basis (Triandis, 2001). It would be interesting for future research to see how those who apply an individualistic orientation may view societal problems, and whether they see entrepreneurship as an appropriate means to solve them.

Maximizing has been associated with building more market and entrepreneurially oriented businesses that achieve greater financial success (Soltwisch, 2021); thus, it is possible that an individualistic orientation may shape the way an entrepreneur goes about starting new ventures, perhaps impacting how successful they are depending on the support they receive for their individual efforts. Based on results from the U.S. sample indicating that individualistic maximizers are especially interested in entrepreneurial opportunities, it is conceivable that early in the startup process, an independent minded entrepreneur may be able to break the mold of what is commonly done by turning an idea into a viable product or service venture. In individualistic cultures (vs. collectivistic), people tend to favor charismatic leaders that can bring new ideas to market, and view leaders who have typical leader qualities in high regard (Ensari and Murphy, 2003). For example, Steve Jobs was notorious for his charismatic leadership style that united people around Apple’s most innovative products (Sharma and Grant, 2011). However, as a business grows around new products and services over time, it would be interesting to see how individualism may affect a leader’s ability to get others involved in building a shared vision for their collective efforts. Perhaps individualism is useful during the early stages of taking an idea to market, but a more collective outlook garners greater support as the business matures. It may be fruitful for future research to explore the longitudinal effects of entrepreneurs who apply an individualistic perspective to maximizing their decision efforts to understand these differences in style over time.

In addition to desiring the best results of their decisions, maximizers want to be the best, emphasizing relative outcomes over objective ones (Weaver et al., 2015; Luan et al., 2018). It makes sense that they apply a more individualist view as they prefer to control the decision-making process with the goal of obtaining desirable outcomes. Although as mentioned in previous work on maximizing and satisficing, this feeling of personal control over decisional outcomes can weigh on their evaluation of decisions in a way that produces regret and unhappiness (Schwartz et al., 2002), especially when others have done even better than them (Chan, 2021). For maximizers who apply an individualistic view, these detrimental psychological outcomes may be even more pronounced as they feel personally responsible for their decision outcomes. This would be in line with others who have found that maximizers are even more regretful and unhappy in the U.S., where personal decisions are seen as the primary avenue to achieving success, and happiness is considered on relative terms to those who are doing better perhaps socially or economically (Roets et al., 2012). Thus, those who maximize in the U.S. may be inclined to pursue opportunities at the expense of their own well-being. To follow up on this, future research could investigate the cross-cultural impacts of well-being associated with maximizers who start entrepreneurial ventures.

Although new products and services can evolve from a single idea, entrepreneurship is a collaborative process, and the quality of team interactions is critical for success (Lechler, 2001). The results of the current study suggest that maximizers may be more inclined to start new ventures; however, further research is needed on the nature of founding team compositions to see what the best combination of decision strategies may be. It is possible that maximizing and satisficing are complimentary styles that both assist in the start-up process. Maximizers tend to apply a more innovative mindset by searching, sometimes exhaustively, for better alternatives. Yet, they can fall victim to over analyses in a way that leaves them stuck perfecting an idea rather than getting it out to the market. Because entrepreneurs often learn more by doing (Man, 2012), the satisficing mindset may offer a counterbalance to maximizers tendency to over evaluate, encouraging the team to move forward with what is good enough. This strategy may ultimately allow them to speed up the innovative process by receiving valuable feedback early on. Future research could investigate the combination of maximizing and satisficing decision strategies as they relate to successful innovation and new venture decisions. Similarly, it is possible that a new venture team may need a balance of cultural perspectives to ensure that individual pursuits can be supported by collective efforts. This study takes some important first steps toward understanding how decision styles and cultural orientation may affect individuals’ intentions to start new business ventures. Undoubtedly, these findings provide many new avenues to enrich our understanding of the way entrepreneurs make decisions.

### Practical and theoretical implications

Diversity trainings, as discussed by Triandis and Singelis (1998) and by Dimitrov (2005), can also be useful for all business processes, including understanding the entrepreneurial propensity of individuals for the purposes of increasing innovation and economic success of the enterprise. It appears that those who maximize their decisions may show greater entrepreneurial intentions. Another implication of the current study is to identify those who may be inclined to serve social purposes through entrepreneurial means. It appears that, at least in the U.S., those who see additional opportunities through a maximizing decision style may be more inclined to solve social problems through entrepreneurial means. Additionally, those who are less individualistic appear to be more apt toward social entrepreneurship than those who are more individualistic. These traits could be useful in identifying and training those who may promote economic development through entrepreneurial means. For example, a maximizing inventory could be used in entrepreneurial programs to see who may be alert to new business opportunities through information search.

It is possible that maximizing and satisficing are complimentary styles, and both assist in the start-up process. New venture teams may need a balance of cultural perspectives to ensure that individual pursuits can be supported by collective efforts. Furthermore, decision styles could be an indicator of innovative potential, and HR managers could identify employees who may share this mindset to allow for new products and services to meet market needs and fulfill social missions.

### Study limitations

Although this paper explores some important potential antecedents of entrepreneurial behavior, it has some limitations that should be addressed. First, as with many entrepreneurial studies, intentions to start new business ventures may serve as a proxy for entrepreneurial behavior, these intentions do not always predict behavior. A longitudinal study following-up with intentions would lend validity to the model while providing additional insights into how entrepreneurs approach the decision to start new ventures. Although students can be an appropriate sample to measure entrepreneurial intentions because they are at the career decision stage, it would enhance generalizability if the proposed relationships could be tested among working populations. Overall, women are less likely to become entrepreneurs than men (Shane, 2008). Although gender was controlled for in both studies, the uneven number of males (45% in study 1) and females (70%) in study two may account for some of the variations in entrepreneurial intentions, potentially overestimating intentions in the U.S. sample while underestimating in the Slovenian sample. Given the exploratory nature of the research, participants completed all measures in a single survey. Using survey data is common in entrepreneurial studies, however, interesting variations in the research model may be identified if data could be collected using a longitudinal design employing mixed methods. Similarly, common methods variance can be a limitation of such research. As a post-hoc analysis, Harman’s Single Factor Test was far below the 50% threshold recommended by Podsakoff et al. (2003) at 33.82%, suggesting that common methods bias was not a substantial concern in the current study. Finally, although we may begin to identify trends in data across cultures, specific conclusions about national cultural context cannot be drawn from this limited sample. As any exploratory study, replicating these relationships through additional research including other national contexts will enhance generalizability while recognizing boundary conditions for the observed effects.

### Future research directions

It is recommended to explore in the future whether the cultural dimensions are relevant to entrepreneurial innovation. It is also interesting to look deeper into the question - Do export market economies such as the US, in fact, emphasize the individualistic values per Triandis and Gelfand (1998)? Furthermore, it would be intriguing to see how individualism may affect a leader’s ability to get others involved in building a shared vision for their collective efforts over time as a business grows.

Another direction to explore is whether social purposes may be less compelling than other reasons to start new ventures, such as financial gains or desires to be their own boss. A longitudinal study could identify better how maximizers or satisficers navigate the start-up process to see if decision styles and culture impact new venture success together.

It is also possible that the best combination of maximizing and satisficing decision strategies will play together for boosting successful innovation and new venture decisions. The longitudinal effects of entrepreneurs who apply an individualistic perspective to maximizing their decision efforts will also be useful in understanding the different decision-making styles.

## Conclusion

This study takes many first steps in exploring how our decisional preferences of maximizing or satisficing relate to our cultural perspective and our tendencies to launch new business ventures. The findings provide many new avenues to enrich our understanding of the way entrepreneurs make decisions. Specifically, identifying that individual’s decision styles and cultural perspectives shape the way people perceive new business opportunities may help to shape policy and identify individuals who are apt to start new ventures. Despite the global mindset of the business world, there will always be differences between the values and perceptions of people from different parts of the world. This study further identifies that these values should be considered as nations attempt to stimulate economic activity through the development of new business ventures.

## Data availability statement

The raw data supporting the conclusions of this article will be made available by the authors, without undue reservation.

## Ethics statement

The studies involving human participants were reviewed and approved by Institutional Review Board, University of Northern Colorado. The patients/participants provided their written informed consent to participate in this study.

## Author contributions

All authors listed have made a substantial, direct, and intellectual contribution to the work and approved it for publication.

## Conflict of interest

The authors declare that the research was conducted in the absence of any commercial or financial relationships that could be construed as a potential conflict of interest.

## Publisher’s note

All claims expressed in this article are solely those of the authors and do not necessarily represent those of their affiliated organizations, or those of the publisher, the editors and the reviewers. Any product that may be evaluated in this article, or claim that may be made by its manufacturer, is not guaranteed or endorsed by the publisher.
